# Supplementary material for: CBP phosphorylation maintains intestinal homeostasis by supporting the stem cell niche through versican
Source: Nat Commun. 2026 Mar 28;17:4583. doi: 10.1038/s41467-026-71083-x (PMC13195047; doi:10.1038/s41467-026-71083-x)
Supplement: Supplementary file 1 — Supplementary Information [file 41467_2026_71083_MOESM1_ESM.docx]

**Supplementary Information**

**CBP phosphorylation maintains intestinal homeostasis by supporting the stem cell niche through versican**

**Authors:** Yi-Ting Lin^1^, Chi Liu^1^, Yu-Hua Hsu^2^, Shi-Chuen Miaw^2^, Ming-Shiang Wu^3^, and Ching-Chow Chen^1*^

***Corresponding author**

^1^Department of Pharmacology and ^2^Graduate Institute of Immunology, College of Medicine, National Taiwan University, Taipei, 100233, Taiwan; ^3^Department of Internal Medicine, National Taiwan University Hospital, Taipei, 100225, Taiwan

**Correspondence** **to:** Dr. Ching-Chow Chen, [chingchowchen@ntu.edu.tw](mailto:chingchowchen@ntu.edu.tw)

**Inventory of Supplementary Information**

Supplementary Table 1

Supplementary Table 2

Supplementary Figure 1, related to Fig. 1

Supplementary Figure 2, related to Fig. 2

Supplementary Figure 3, related to Fig. 2

Supplementary Figure 4, related to Fig. 4

Supplementary Figure 5, related to Fig. 5

Supplementary Figure 6, related to Fig. 7

Supplementary Figure 7, related to Fig. 4

Supplementary Figure 8, related to Fig. 5 and 8

Supplementary Figure 9, related to Supplementary Figure 2g

Supplementary Figure 10, related to Supplementary Figure 2h

Supplementary Figure 11, related to Supplementary Figure 7a

**Supplementary Table**

**Supplementary Table 1 Statistical results of genotyping in *CBP^AA^* mice.**

|  | +/m x +/m | | |
| --- | --- | --- | --- |
| Genotype of offspring | WT | +/m | AA |
| Born | 62 | 100 | 42 |
| Ratio | 1 | 1.61 | 0.68 |
| Expected ratio | 1 | 2 | 1 |

**Supplementary Table 2 Primers used for plasmid construction and sequencing.**

| Primer Name | Sequence (5' to 3') | Usage / Target |
| --- | --- | --- |
| BMRC-6585 | gggcggccgggaattcATGGCCGAGAACTTGCTGGA | pCMV-puro-IRES2-EGFP_mCBP (EcoRI) |
| BMRC-6262 | GCTATGACCgcggccgcTTTACTTGTACAGCTCGTC | pCMV-puro-IRES2-EGFP_mCBP (NotI) |
| BMRC-6258 | AGATTCgtcCATCTCTCCAtcATCCACAAACCTTGACTTCA | mCBP S1383D S1387D (DD) |
| BMRC-6587 | GAGATGgacGAATCTTTCCCATATCGTACCA | mCBP S1383D S1387D (DD) |
| BMRC-6591 | AGATTCCtcCATCTCTCCttcATCCACAAACCTTGACTTCA | mCBP S1383E S1387E (EE) |
| BMRC-6264 | GAGATGgaGGAATCTTTCCCATATCGTACCA | mCBP S1383E S1387E (EE) |
| BMRC-6260 | AGTCAGGCACGTCGTATGGGTAGGATCCCAAACCCTCCACAAACTTTTC | IRES2-GFP |
| BMRC-6589 | ACGACGTGCCTGACTACGCCTAGGAATTCTGCAGTCGACGGTACCGCG | IRES2-GFP |
| BMRC-6593 | GAGGCATGACCAAGATGG | for DNA sequencing (on mCBP) |
| BMRC-6595 | GGGGCTACCAATCCACTG | for DNA sequencing (on mCBP) |
| BMRC-6597 | AACCCTCTGAACATGCTG | for DNA sequencing (on mCBP) |
| BMRC-6599 | CTCCTAGGAATCCCAGAT | for DNA sequencing (on mCBP) |
| BMRC-6601 | TCTCTTCCTTACGCCGCT | for DNA sequencing (on mCBP) |

**Supplementary Figures**

**
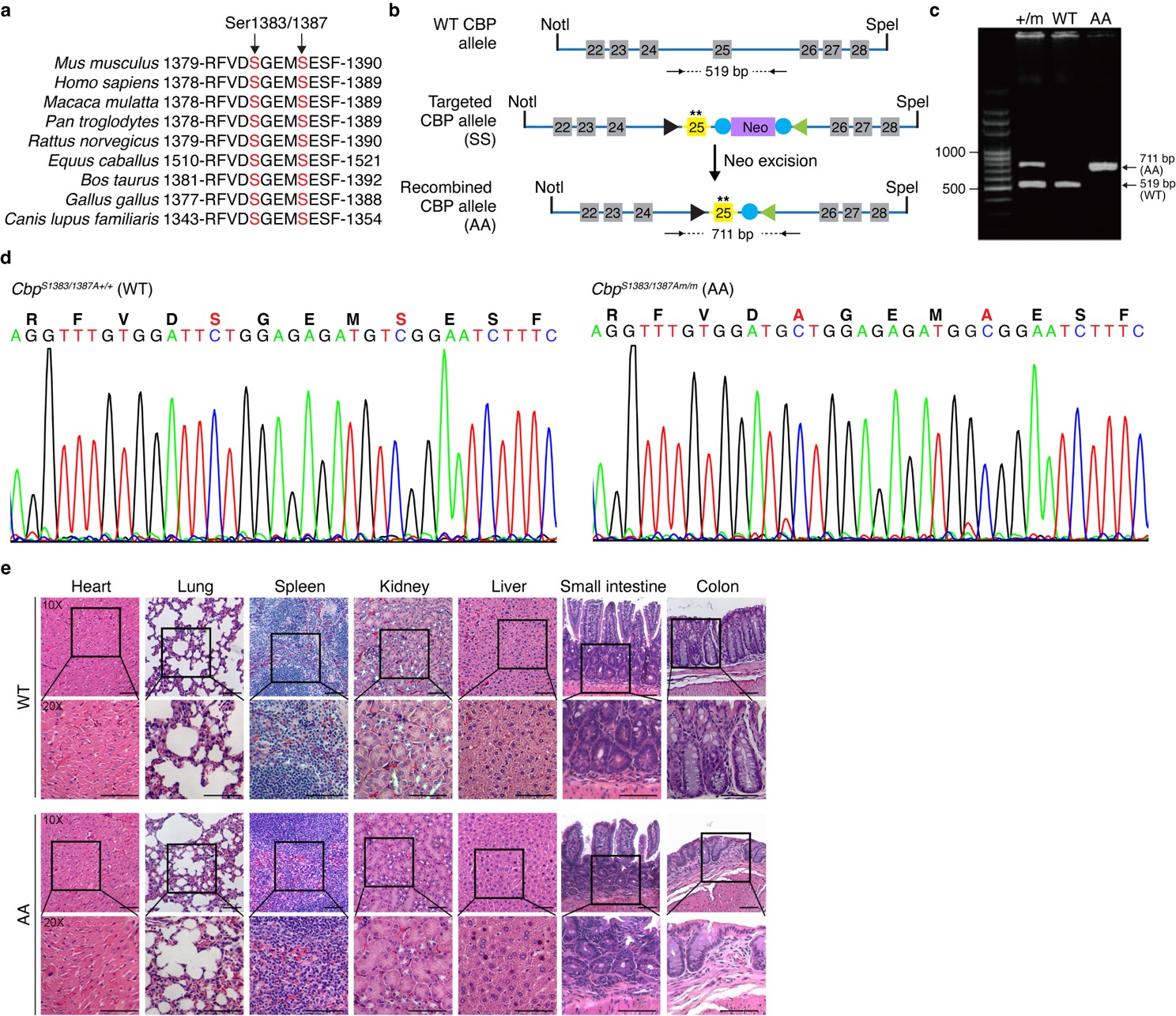
Supplementary Figure 1** **CBP phosphorylation sites, targeting strategy, genotyping and H&E in conventional *CBP^AA^* knock-in mice.** **a** CBP phosphorylation sites at Ser1383/1387 (in mice) are conserved across eukaryotic species. **b** Targeting strategy for conventional *CBP^AA^* knock-in mice (*CBP^AA^* mice). **c, d** Genomic DNA extracted from the tails of *CBP^WT^* and *CBP^AA^* mice. The WT and AA alleles were amplified by PCR (**c**). Molecular weight markers are indicated in base pairs (bp). The PCR products were purified, and mutant bases were confirmed by sequencing (**d**). Chromatograms show the confirmed codons for Alanine (A) in SSAA mutants compared to Serine (S) in WT. **e** All organ sections were counterstained with hematoxylin and eosin (H&E), and high-magnification images of the areas enclosed by black outlines are shown. Representative images from n = 3 mice per genotype with similar results are shown. Scale bars, 100 μm. Source data are provided as a Source Data file.

**
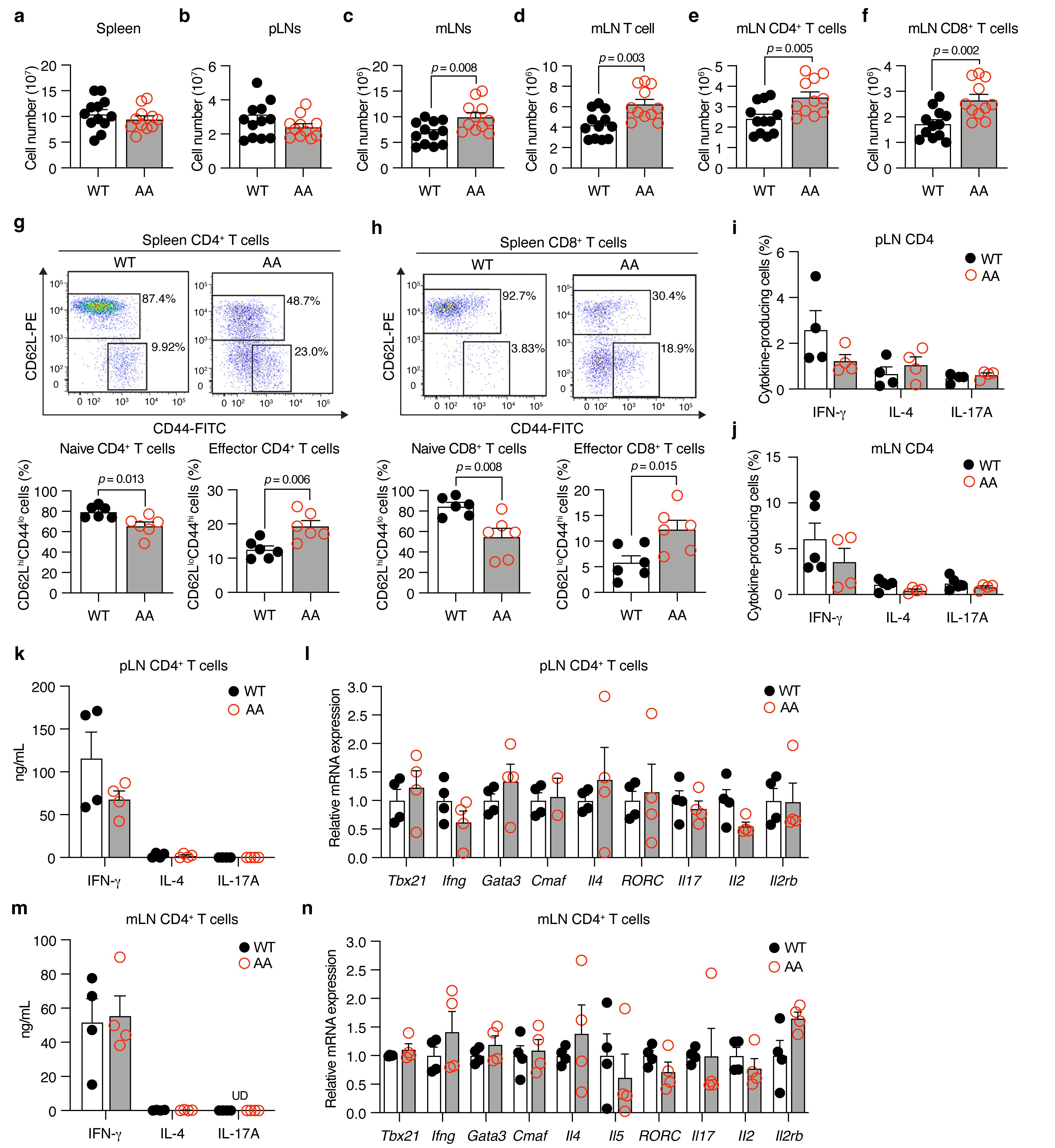
Supplementary Figure 2 Impaired CBP phosphorylation does not interfere immune cells.** Absolute cell numbers in the spleen (**a**), peripheral lymph nodes (pLNs) (**b**), and mesenteric lymph nodes (mLNs) (**c**) from *CBP^WT^* and *CBP^AA^* mice. **d**-**f** Cellularity of T cells (**d**), CD4^+^ T cells (**e**), and CD8^+^ T cells (**f**) in mLNs, analyzed by flow cytometry. n = 12 mice for *CBP^WT^*, n = 11 mice for *CBP^AA^*. **g**, **h** Percentage of naïve (CD62L^hi^CD44^lo^) and effector/memory T cells (CD62L^lo^CD44^hi^) among CD4^+^ **(g)** and CD8^+^ **(h)** populations in the spleen. n = 6 mice per group. For the detailed sequential gating strategy, see Supplementary Figure 9 and 10. **i**-**n** Functional analysis of sorted CD4^+^ T cells from pLNs and mLNs. T cells were isolated via flow cytometry (CD4^+^TCR-β^+^), cultured with anti-CD3 (1 μg/mL), anti-CD28 (1 μg/mL), and IL-2 (100 U/mL) for 4 days, and restimulated with PMA (50 ng/mL) and ionomycin (500 ng/mL) for 3 h prior to flow cytometric analysis **(i, j)**. For cytokine and gene expression assays **(k–n)**, cells were restimulated with anti-CD3 (1 μg/mL) for 24 h to collect supernatants for ELISA or processed for qPCR analysis. n = 4 mice per group. Data are presented as mean ± SEM. All *p* values were calculated using unpaired two-tailed Student’s *t*-test and are indicated on the graphs. Source data are provided as a Source Data file.

**
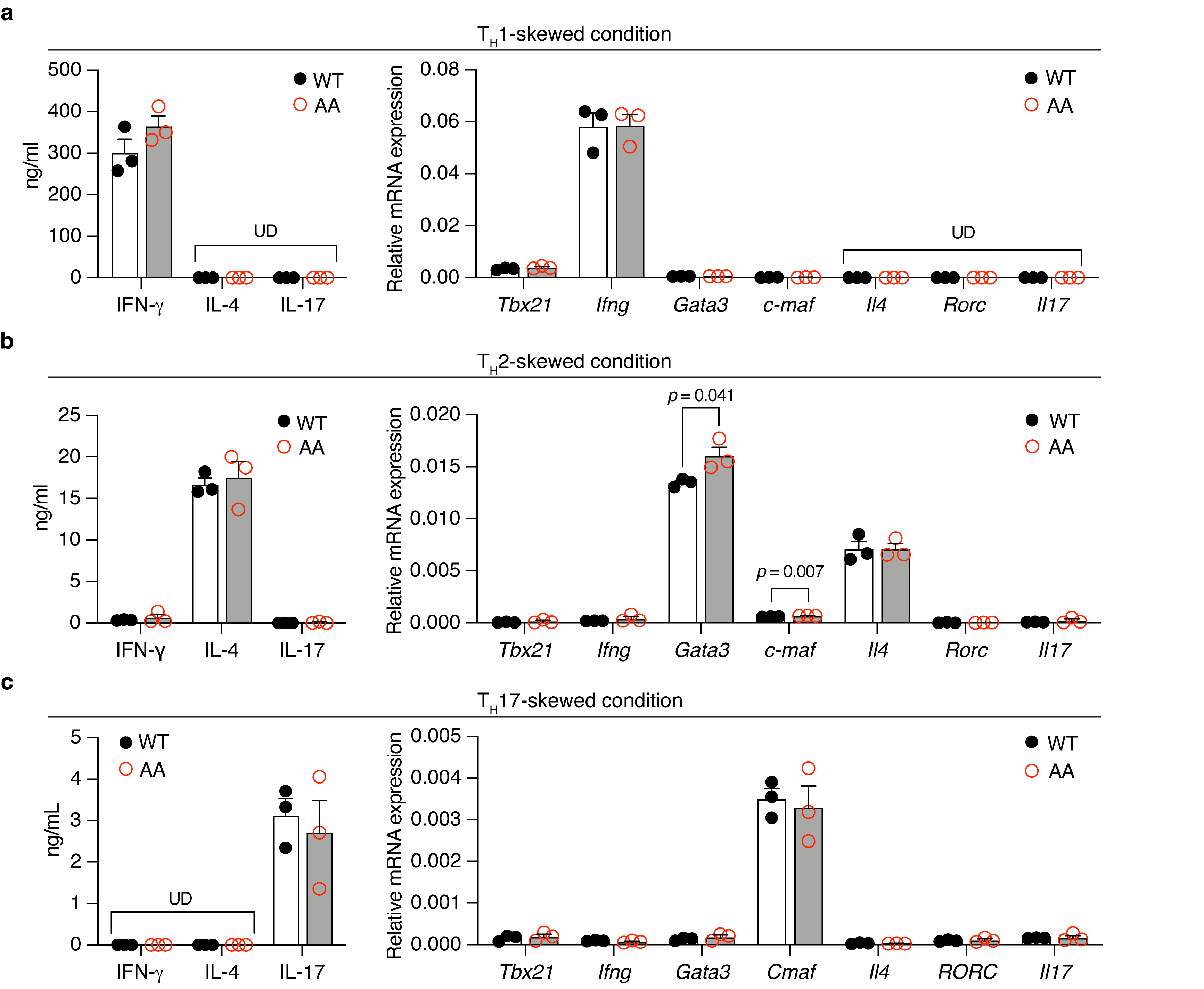
**

**Supplementary Figure 3 Impaired CBP phosphorylation does not interfere differentiation potential of T_H_ subsets. a**-**c** Naïve CD4^+^ T cells (CD62L^hi^CD44^lo^) sorted from the peripheral lymph nodes of *CBP^WT^* and *CBP^AA^* mice were cultured under the T_H_1 (**a**), T_H_2 (**b**) or T_H_17 (**c**) skewing conditions for 4 days. The skewing recipes were as follows: T_H_1: IL-12 (1 ng/mL), IL-2 (100 U/mL), and anti-IL-4 (10 μg/mL); T_H_2: IL-4 (10 ng/mL), IL-2 (100 U/mL), and anti-IFN-γ (10 μg/mL); T_H_17: IL-6 (20 ng/mL), TGF-β (2.5 ng/mL), IL-23 (20 ng/mL), soluble anti-CD28 (2 μg/mL), anti-IFN-γ (10 μg/mL), and anti-IL-4 (10 μg/mL). All conditions used wells coated with anti-CD3ε (1 μg/mL) for primary stimulation, with T_H_1 and T_H_2 conditions supplemented with soluble anti-CD28 (1 μg/mL). Following differentiation, cells were restimulated with anti-CD3ε (1 μg/mL) for 24 h. Cytokine production in supernatants and mRNA expression levels were analyzed by ELISA and qPCR, respectively. Gene expression levels were normalized to β-actin. n = 3 mice per group. Data are presented as mean ± SEM. All *p* values were calculated using unpaired two-tailed Student’s *t*-test and are indicated on the graphs. UD, undetectable. Source data are provided as a Source Data file.

**
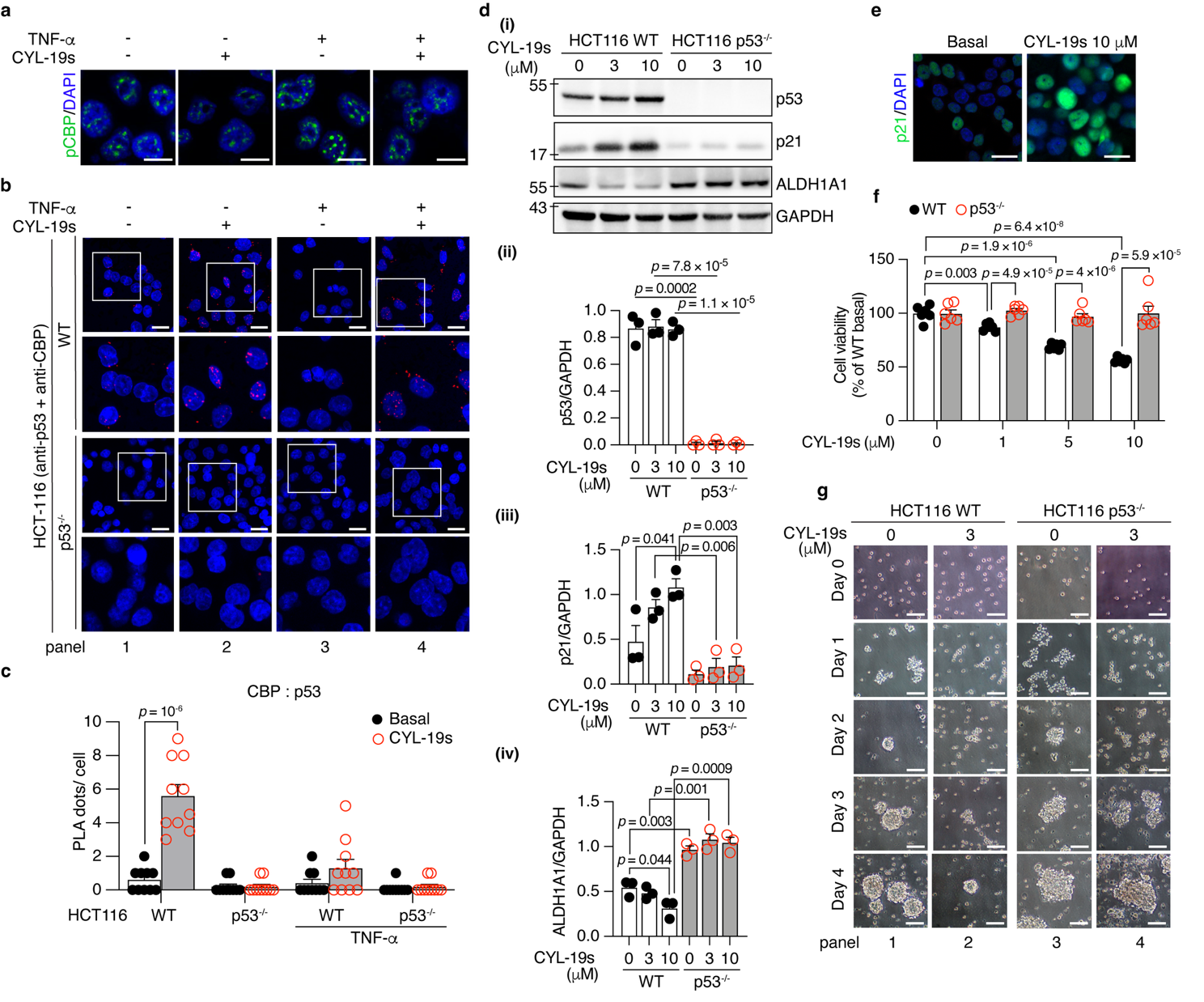
**

**Supplementary Figure 4 Impaired phosphorylation of CBP reduces spheroid formation in HCT116 cells.** HCT116 WT and *p53^-/-^* cells were treated with CYL-19s at indicated concentrations for 24 hours, followed by treatment with or without 10 ng/mL TNF-α for an additional 2 hours. **a** Representative immunofluorescence images of phospho-CBP (pCBP) staining. **b**, **c** Representative images (**b**) and quantification (**c**) of PLA detecting the association between CBP and p53. Nuclei were counterstained with DAPI to identify individual cells. Quantification of PLA signals (dots) per cell. For each group, 10 cells were randomly selected for quantification using ImageJ. Scale bars, 20 μm. **d** Representative immunoblots (**i**) and quantification (**ii**-**iv**) of the indicated proteins in total lysates. Band intensities were quantified using ImageJ and normalized to GAPDH, which served as a loading control. n = 3 independent biological replicates. **e** Representative immunofluorescence staining of p21 in HCT116 cells. Nuclei were counterstained with DAPI. Scale bars, 20 μm. **f** Cell viability measured by WST-1 assay. Viability was normalized to HCT116 WT cells treated with 0 μM CYL-19s (basal). n = 6 independent biological replicates. **g** Representative images of sphere formation from day 1 to day 4. Scale bars, 100 μm. Results for **a–c**, **e**, and **g** are representative of n = 3 independent experiments with similar results. Data are presented as mean ± SEM. All *p* values were calculated using unpaired two-tailed Student’s *t*-test and are indicated on the graphs. Source data are provided as a Source Data file.


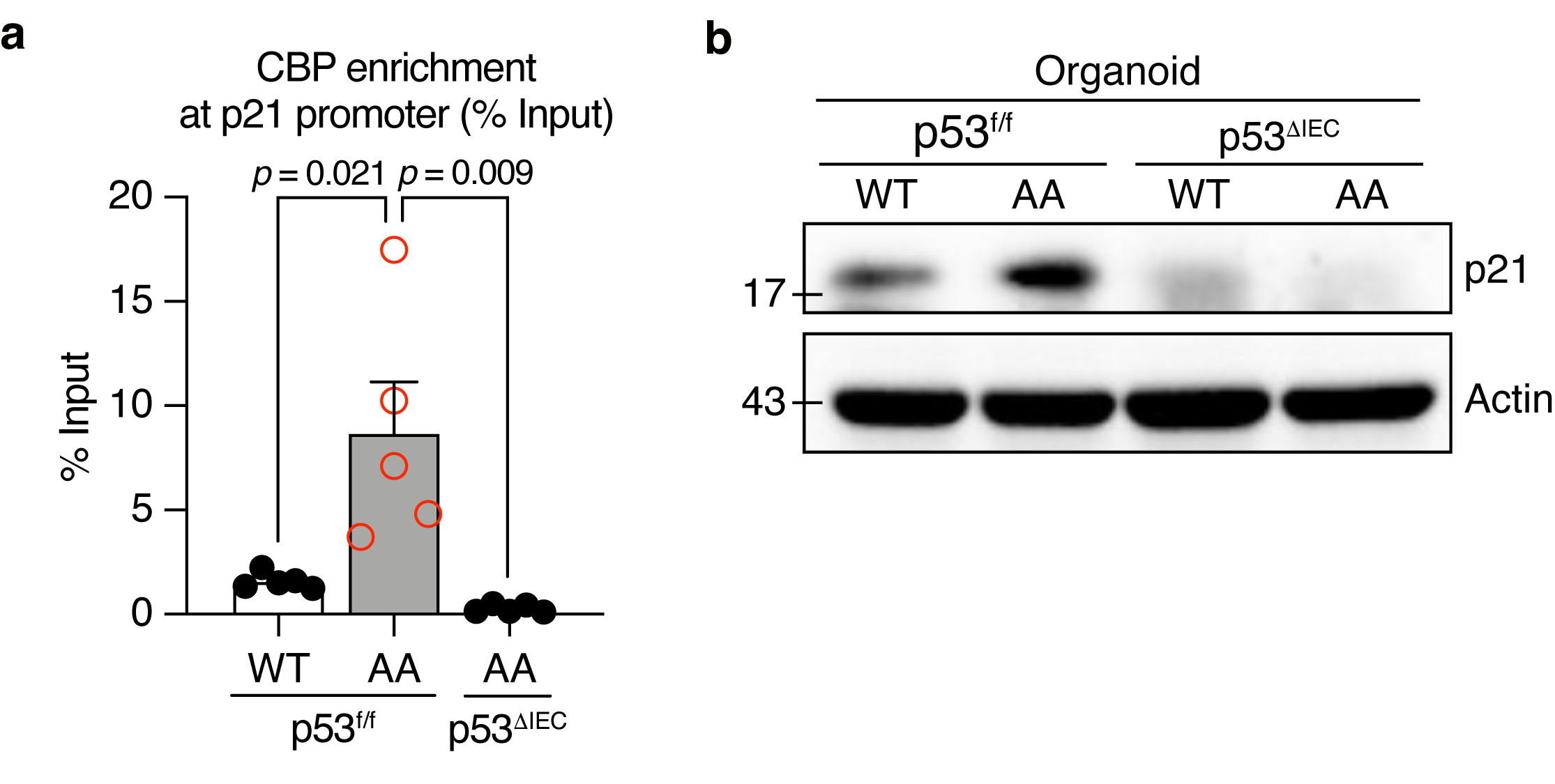


**Supplementary Figure 5 Direct regulation of CBP on p53 is enhanced at the p21 promoter to mediate gene expression in *CBP^AA^* mice. a** Enrichment of CBP at the p21 (*Cdkn1a*) promoter in isolated colonic crypts from *CBP^WT^p53^f/f^*, *CBP^AA^p53^f/f^* and *CBP^AA^p53^ΔIEC^* mice, measured by CUT&RUN assay using anti-CBP antibody. Data are presented as % input normalized to the input DNA of each sample. n = 5 mice per group. **b** Immunoblots showing p21 levels in total lysates from cultured organoids on Day 6. β-Actin served as a loading control. Data from a single experiment are presented due to limited sample availability from organoid cultures. Data are presented as the means ± SEMs. All *p* values were calculated using an unpaired two-tailed Student’s *t* test and are indicated on the graphs. Source data are provided as a Source Data file.


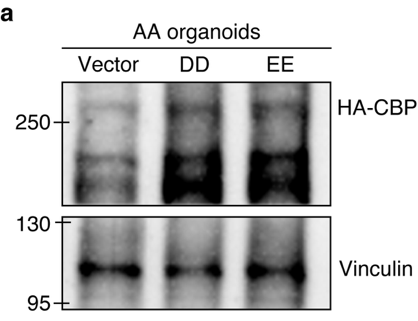


**Supplementary Figure 6 Expression of phospho-mimetic CBP mutants in *CBP^AA^p53^f/f^* organoids.** Western blot analysis of HA-tagged CBP expression in day 5 cultured *CBP^AA^p53^f/f^* organoids transfected with pCMV-puro-IRES2-*EGFP* (vector), pCMV-puro-IRES2-*EGFP mCBP^S1383D/S1387D^*-HA (DD), or pCMV-puro-IRES2-*EGFP mCBP^S1383E/S1387E^*-HA (EE) using Lipofectamine. The presence of HA-CBP (DD and EE mutants) was detected using an anti-HA antibody to verify protein expression. Vinculin served as a loading control. Data from a single experiment are shown due to limited sample availability from transfected organoids. Source data are provided as a Source Data file.

**
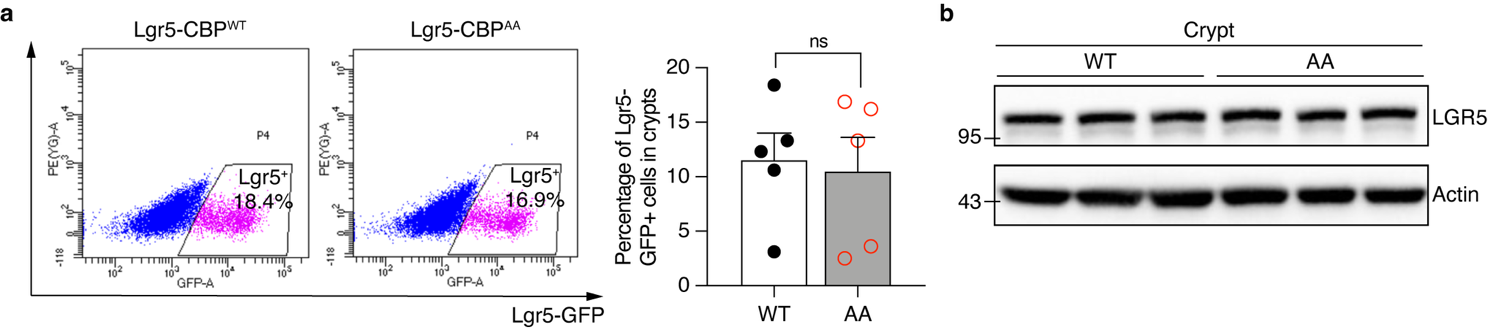
**

**Supplementary Figure 7 No alteration in Lgr5^+^ stem cells between *CBP^WT^* and *CBP^AA^* mice. a** Representative flow cytometry plots and quantification of GFP^+^ signal in isolated and dissociated colonic crypt cells from *Lgr5-eGFP-creERT2^+/Tg^; Villin-Cre; CBP^WT^* (*Lgr5-CBP^WT^*) and *Lgr5-eGFP-creERT2^+/Tg^; Villin-Cre; CBP^AA^* (*Lgr5-CBP^AA^*) mice. For the detailed sequential gating strategy, see Supplementary Figure 11. n = 5 mice per group. **b** Representative immunoblots showing LGR5 expression in total lysates of colonic crypts from *CBP^WT^* and *CBP^AA^* mice. β-actin was used as the internal control. n = 3 mice per group. Data are presented as the mean ± SEM. All *p* values were calculated using unpaired two-tailed Student’s t-test. ns, not significant. Source data are provided as a Source Data file.

**
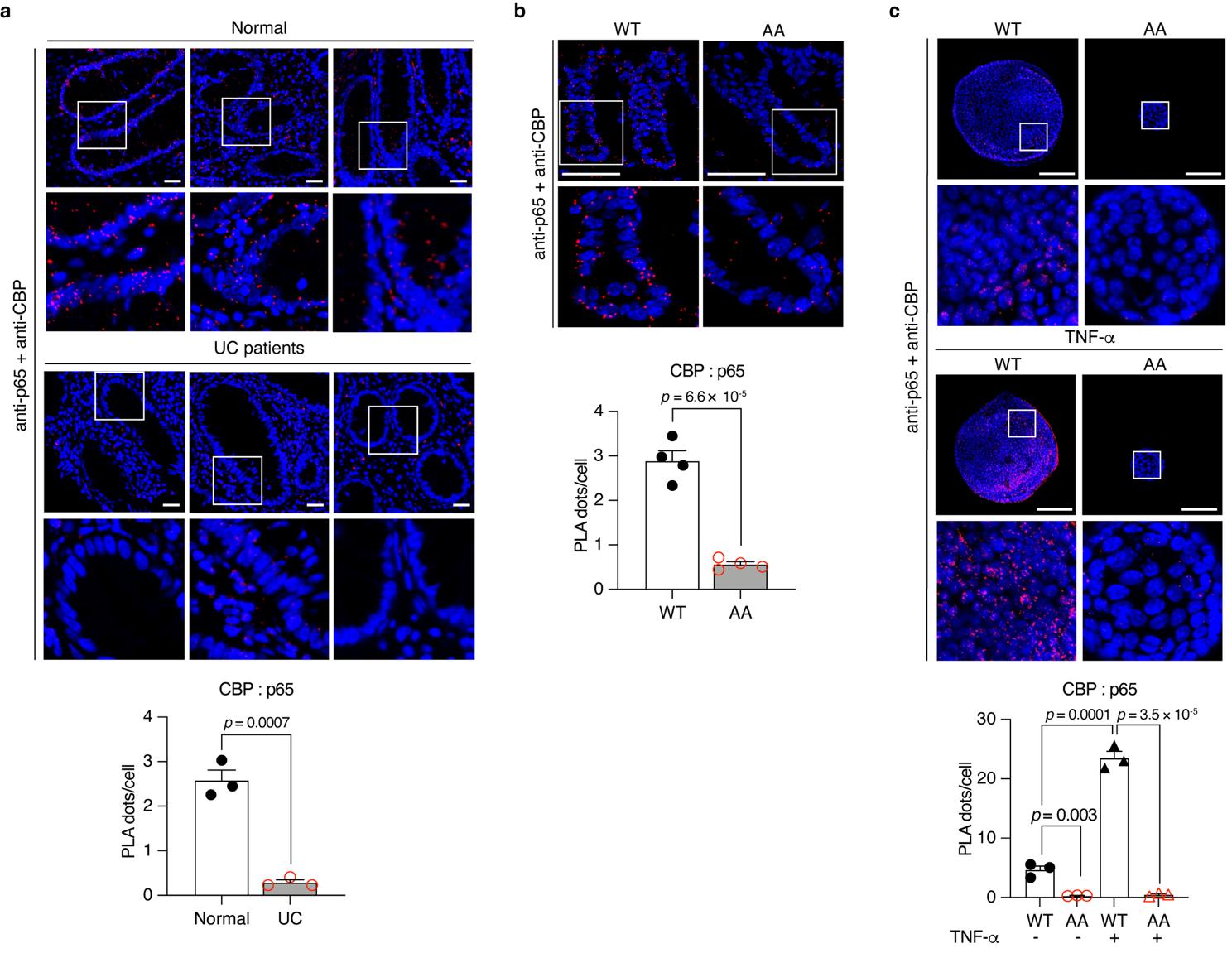
**

**Supplementary Figure 8 Detection of CBP-p65 interaction by proximity ligation assay (PLA). a**-**c** Representative images and quantification of PLA detecting CBP–p65 association in colon sections from healthy controls and UC patients (**a**), *CBP^WT^* and *CBP^AA^* mice (**b**), and day 6 cultured *CBP^WT^p53^f/f^* and *CBP^AA^p53^f/f^* organoids treated with or without 50 ng/mL mouse TNF-α for 2 hours (**c**). Nuclei were counterstained with DAPI and used to identify individual cells. PLA signals (red dots) per cell were quantified using ImageJ. (**a**, **c**) n = 3 biological replicates per group; (**b**) n = 4 mice per group. Data are presented as mean ± SEM. All *p* values were calculated using unpaired two-tailed Student’s *t*-test and are indicated on the graphs. Scale bars: 20 μm (**a**), 50 μm (**b**), 100 μm (**c**). Source data are provided as a Source Data file.

**
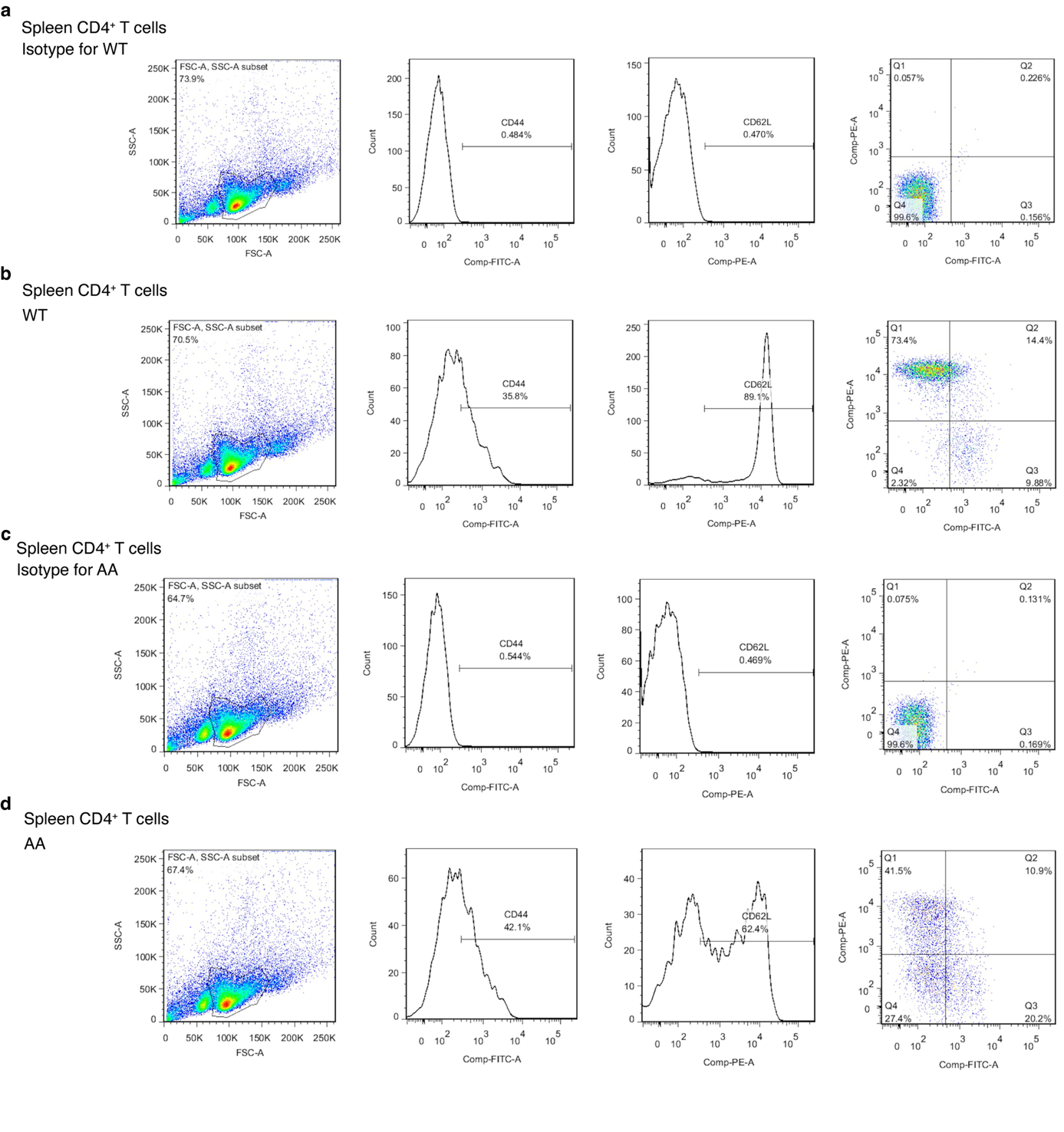
Supplementary Figure 9 Original flow cytometry analysis and isotype control verification** **for splenic CD4^+^ T cells.** Sequential gating hierarchy and control setting for the characterization of CD44 and CD62L expression in splenic CD4^+^ T cells. **(a, c)** Isotype controls: **(a)** *CBP^WT^* and **(c)** *CBP^AA^* background controls. Isotype-matched antibodies were used to define the fluorescence thresholds for CD44 and CD62L and to set the quadrant gates, ensuring accurate differentiation between specific signal and background noise. **(b, d)** Representative experimental groups: Gating logic for **(b)** *CBP^WT^* and **(d)** *CBP^AA^* samples. Gating hierarchy: cell identification: Total splenic cells were first identified and gated based on their size and granularity (FSC-A vs. SSC-A) to exclude debris. Marker definition: histograms (middle panels) show the distribution of CD44-FITC and CD62L-PE intensity within the gated CD4^+^ population. Quadrant analysis: final dot plots (right panels) illustrate the co-expression of CD44 and CD62L. Gates were consistently applied across all samples based on the boundaries established by the isotype controls. Note on gating refinement: Supplementary Figure 2g were refined from these raw quadrant gates into rectangular gates for precise cluster isolation. Data and quantification logic remain identical and are based on the isotype-defined thresholds shown here.

**
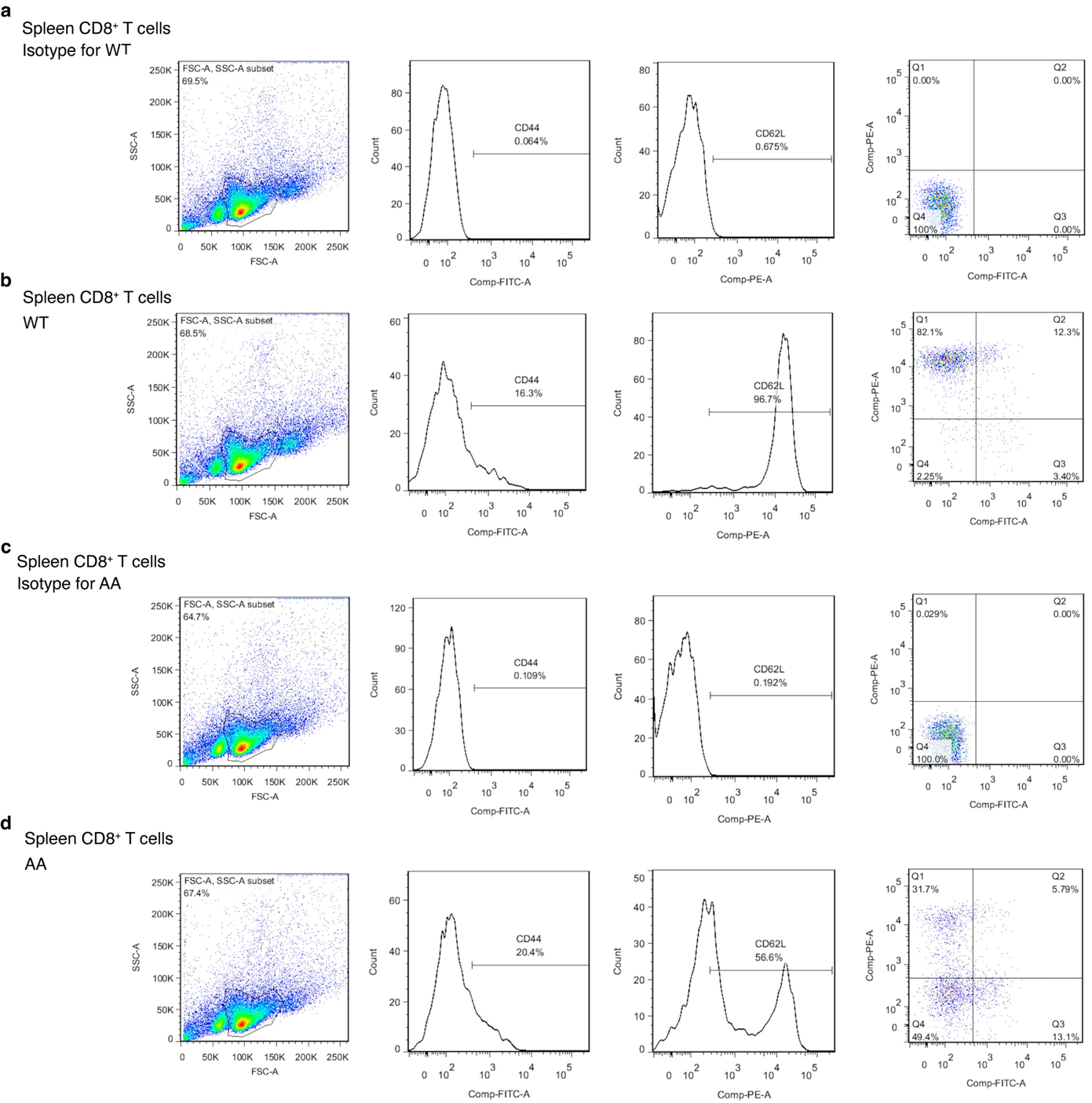
Supplementary Figure 10 Original flow cytometry analysis and isotype control verification for splenic CD8^+^ T cells.** Sequential gating hierarchy and control setting for the characterization of CD44 and CD62L expression in splenic CD8^+^ T cells. **(a, c)** Isotype controls: **(a)** *CBP^WT^* and **(c)** *CBP^AA^* background controls. Isotype-matched antibodies were used to define the fluorescence thresholds for CD44 and CD62L and to set the quadrant gates, ensuring accurate differentiation between specific signal and background noise. **(b, d)** Representative experimental groups: Gating logic for **(b)** *CBP^WT^* and **(d)** *CBP^AA^* samples. Gating hierarchy: cell identification: Total splenic cells were first identified and gated based on their size and granularity (FSC-A vs. SSC-A) to exclude debris. Marker definition: histograms (middle panels) show the distribution of CD44-FITC and CD62L-PE intensity within the gated CD8^+^ population. Quadrant analysis: final dot plots (right panels) illustrate the co-expression of CD44 and CD62L. Gates were consistently applied across all samples based on the boundaries established by the isotype controls. Note on gating refinement: Supplementary Figure 2h were refined from these raw quadrant gates into rectangular gates for precise cluster isolation. Data and quantification logic remain identical and are based on the isotype-defined thresholds shown here.

**
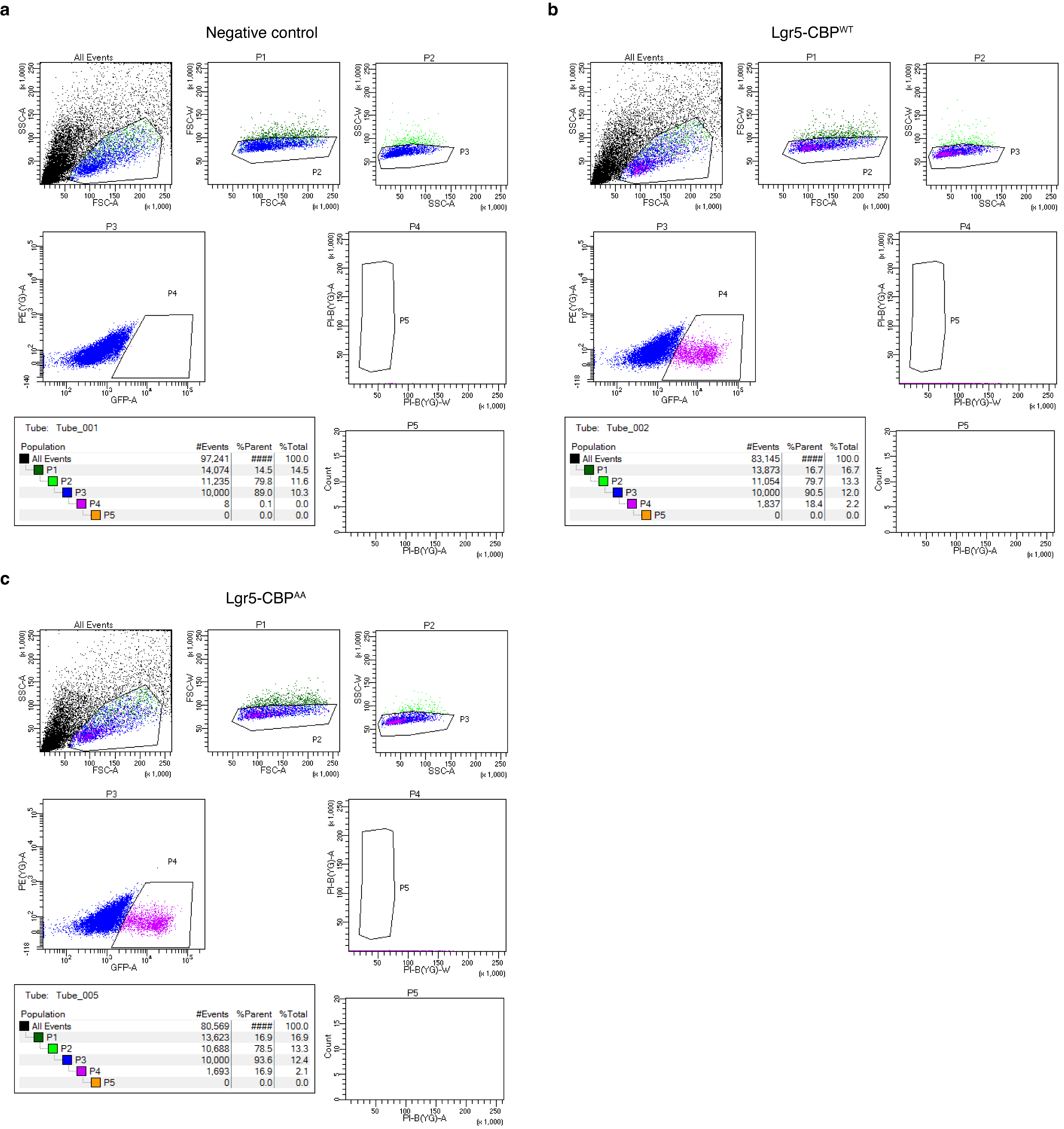
Supplementary Figure 11 Representative gating strategy for colonic Lgr5-GFP^+^ stem cell isolation.** Sequential gating hierarchy used to identify and isolate Lgr5-GFP^+^ stem cells from colonic crypts. **a** Gating strategy for the negative control: Samples from *Villin-Cre; CBP^WT^* mice (without the *Lgr5-eGFP* reporter) were used to define background autofluorescence and establish the threshold for the GFP^+^ population (P4). **b, c** Representative gating for experimental groups: Gating logic for **(b)** *Lgr5-eGFP-creERT2^+/Tg^; Villin-Cre; CBP^WT^* (*Lgr5-CBP^WT^*) and **(c)** *Lgr5-eGFP-creERT2^+/Tg^; Villin-Cre; CBP^AA^* (*Lgr5-CBP^AA^*) samples. Sequential hierarchy: P1 (cells): identification of the total cell population based on FSC-A vs. SSC-A to exclude debris. P2 & P3 (doublet exclusion): sequential gating using FSC-W vs. FSC-A (P2) and SSC-W vs. SSC-A (P3) to ensure single-cell analysis and exclude cell aggregates. P4 (Lgr5-GFP^+^ selection): selection of the GFP-high population representing intestinal stem cells. P5 (population refinement): final gate applied to ensure the morphological homogeneity of the sorted Lgr5-GFP^+^ cells. Percentage values within the plots indicate the proportion of events relative to the parent gate.
