# Supplementary material for: CBP phosphorylation maintains intestinal homeostasis by supporting the stem cell niche through versican
Source: Nat Commun. 2026 Mar 28;17:4583. doi: 10.1038/s41467-026-71083-x (PMC13195047; doi:10.1038/s41467-026-71083-x)
Supplement: Supplementary file 2 — Reporting Summary [file 41467_2026_71083_MOESM2_ESM.pdf]

nature portfolio

Corresponding author(s): Ching-Chow Chen

Last updated by author(s): Mar 6, 2026

Reporting Summary

Nature Portfolio wishes to improve the reproducibility of the work that we publish. This form provides structure for consistency and transparency in reporting. For further information on Nature Portfolio policies, see our Editorial Policies and the Editorial Policy Checklist.

Statistics

For all statistical analyses, confirm that the following items are present in the figure legend, table legend, main text, or Methods section.

n/a	Confirmed	
☐	☒	The exact sample size (*n*) for each experimental group/condition, given as a discrete number and unit of measurement
☐	☒	A statement on whether measurements were taken from distinct samples or whether the same sample was measured repeatedly
☐	☒	The statistical test(s) used AND whether they are one- or two-sided<br>*Only common tests should be described solely by name; describe more complex techniques in the Methods section.*
☒	☐	A description of all covariates tested
☐	☒	A description of any assumptions or corrections, such as tests of normality and adjustment for multiple comparisons
☐	☒	A full description of the statistical parameters including central tendency (e.g. means) or other basic estimates (e.g. regression coefficient) AND variation (e.g. standard deviation) or associated estimates of uncertainty (e.g. confidence intervals)
☐	☒	For null hypothesis testing, the test statistic (e.g. *F*, *t*, *r*) with confidence intervals, effect sizes, degrees of freedom and *P* value noted<br>*Give P values as exact values whenever suitable.*
☒	☐	For Bayesian analysis, information on the choice of priors and Markov chain Monte Carlo settings
☒	☐	For hierarchical and complex designs, identification of the appropriate level for tests and full reporting of outcomes
☒	☐	Estimates of effect sizes (e.g. Cohen's *d*, Pearson's *r*), indicating how they were calculated

Our web collection on statistics for biologists contains articles on many of the points above.

Software and code

Policy information about availability of computer code

Data collection	• Confocal imaging: ZEN software (Carl Zeiss) for LSM 780 and LSM 880 confocal microscopes.<br>• Whole-slide imaging: MicroVisioneer Manual Whole-Slide Imaging Software and TissueFAXS software (TissueGnostics GmbH).<br>• Flow cytometry: BD FACSDiva software (used for data acquisition on FACSAria III and LSRFortessa).<br>• Real-time PCR: QuantStudio Design and Analysis Software (Thermo Fisher Scientific).<br>• Plate reader: Paradigm Detection Platform software (Beckman Coulter) and Gen5 software (BioTek).
Data analysis	• Image analysis: ImageJ2/Fiji (v2.16.0/1.54p).<br>• Statistical analysis: GraphPad Prism 10 (v10.2.3).<br>• Flow cytometry analysis: FlowJo software was used for T cell population analysis; BD FACSDiva software was used for Lgr5+ cell gating and analysis.<br>• RNA-seq & Bioinformatics: Standard bioinformatics pipelines provided by Genomics BioSci & Tech (New Taipei City, Taiwan); SR plot and Venny 2.1 for data visualization.

For manuscripts utilizing custom algorithms or software that are central to the research but not yet described in published literature, software must be made available to editors and reviewers. We strongly encourage code deposition in a community repository (e.g. GitHub). See the Nature Portfolio guidelines for submitting code & software for further information.

Data

Policy information about availability of data

All manuscripts must include a data availability statement. This statement should provide the following information, where applicable:

- Accession codes, unique identifiers, or web links for publicly available datasets
- A description of any restrictions on data availability
- For clinical datasets or third party data, please ensure that the statement adheres to our policy

The RNA sequencing data generated in this study have been deposited in the Gene Expression Omnibus (GEO) database under accession code GSE295556. The processed figures and source data are available in the Figshare database under https://doi.org/10.6084/m9.figshare.28837331. The raw confocal immunofluorescence images are protected and are not available in a public repository due to data privacy laws, as the metadata contains non-de-identifiable patient identifiers; however, these data are available from the corresponding author upon request. The source data generated in this study are provided in the Source Data file. Source data are provided with this paper.

Research involving human participants, their data, or biological material

Policy information about studies with human participants or human data. See also policy information about sex, gender (identity/presentation), and sexual orientation and race, ethnicity and racism.

Reporting on sex and gender	No individual-level data was provided in this study and thus sex/gender was not reported.
Reporting on race, ethnicity, or other socially relevant groupings	N/A
Population characteristics	Paraffin-embedded colon sections from healthy controls and patients diagnosed with Ulcerative Colitis (UC) were used for IHC, IF, and PLA staining as described in the Methods and Figure Legends.
Recruitment	Human tissue samples were obtained from archived paraffin-embedded blocks at National Taiwan University Hospital. Informed consent was obtained from all participants prior to sample collection.
Ethics oversight	The use of human paraffin-embedded colon sections was approved by the Institutional Review Board (IRB) of National Taiwan University Hospital (Nos. 201705031RIND and 202404149RINC).

Note that full information on the approval of the study protocol must also be provided in the manuscript.

Field-specific reporting

Please select the one below that is the best fit for your research. If you are not sure, read the appropriate sections before making your selection.

- [x] Life sciences
- [ ] Behavioural & social sciences
- [ ] Ecological, evolutionary & environmental sciences

For a reference copy of the document with all sections, see nature.com/documents/nr-reporting-summary-flat.pdf

Life sciences study design

All studies must disclose on these points even when the disclosure is negative.

Sample size	All experiments reported in this study were repeated at least three independent times. For western blotting, immunohistochemistry, immunofluorescence, and H&E, representative images were derived from at least three animals per genotype (mice) or human individuals, as specified in the corresponding figure legends.
Data exclusions	No data were excluded from the analysis.
Replication	Data represent independent biological replicates as indicated in the Materials and Methods and Figure legends. For cell-based and organoid assays, results are derived from at least three independent experiments with similar results, except where otherwise noted in the figure legends (e.g., Supplementary Figure 5b and 6, which provide representative results from a single experiment).
Randomization	For animal studies, mice were randomly assigned to experimental groups. For cell-based assays, samples were allocated into groups randomly.
Blinding	Blinding was performed for clinical scoring, colon length measurements, and histological evaluations by observers or a pathologist blinded to the experimental groups. For biochemical assays (e.g., western blot, qPCR), blinding was not possible due to limited personnel.

Reporting for specific materials, systems and methods

We require information from authors about some types of materials, experimental systems and methods used in many studies. Here, indicate whether each material, system or method listed is relevant to your study. If you are not sure if a list item applies to your research, read the appropriate section before selecting a response.

Materials & experimental systems

n/a	Involved in the study
	☒ Antibodies
	☒ Eukaryotic cell lines
☒	☐ Palaeontology and archaeology
	☒ Animals and other organisms
☒	☐ Clinical data
☒	☐ Dual use research of concern
☒	☐ Plants

Methods

n/a	Involved in the study
☒	☐ ChIP-seq
	☒ Flow cytometry
☒	☐ MRI-based neuroimaging

Antibodies

Antibodies used

Primary Antibodies:
• Anti-pCBP (Ser1383/1387 for mouse; Ser1382/1386 for human): GeneTex, customized, GTX90661; used for IHC (1:100), IF (1:150), and whole-mount organoid staining (1:150).
• Anti-CBP: Cell Signaling Technology, #7389; used for IHC (1:50), IF (1:200), CUT&RUN (1:50), PLA (1:200), and whole-mount organoid staining (1:200).
• Anti-p53: Santa Cruz Biotechnology, sc-126 (IHC 1:100; PLA 1:200; WB 1:2000); Leica, NCL-L-p53-CM5p (WB 1:2000); Cell Signaling Technology, #2524 (CUT&RUN 1:50).
• Anti-p21: Cell Signaling Technology, #2947 (IHC 1:100; IF 1:150; WB 1:1000); Santa Cruz Biotechnology, sc-6246 (WB 1:1000).
• Anti-Ki67: Cell Signaling Technology, #9129S; used for IF (1:200) and whole-mount organoid staining (1:200).
• Anti-E-cadherin: Invitrogen, #33-4000; used for IF (1:200) and whole-mount organoid staining (1:200).
• Anti-versican: Abcam, ab19345; used for IHC (1:100) and IF (1:200).
• Anti-F4/80: Cell Signaling Technology, #70076; used for IHC (1:250).
• Anti-Ly6G: R&D Systems, MAB1037; used for IHC (1:50).
• Anti-IKKα: Santa Cruz Biotechnology, sc-7606; used for IHC (1:100).
• Anti-BrdU: Abcam, ab6326; used for IHC (1:100).
• Anti-p65: Cell Signaling Technology, #6956; used for PLA (1:200).
• Anti-ALDH1A1: Cell Signaling Technology, #12035; used for WB (1:1000).
• Anti-HA-tag: Cell Signaling Technology, #3724; used for WB (1:1000).
• Anti-Lgr5: Abcam, ab75850; used for WB (1:1000).
• Anti-vinculin: Santa Cruz Biotechnology, sc-25336; used for WB (1:1000).
• Anti-GAPDH: Proteintech, #60004-1-Ig; used for WB (1:2000).
• Anti-actin: Proteintech, #66009-1-Ig; used for WB (1:5000).

Flow Cytometry Antibodies:
• Anti-CD45.1 (BUV395): BD Biosciences, #565212; 1:200.
• Anti-CD45.2 (BUV737): BD Biosciences, #612778; 1:200.
• Anti-CD4 (PE): eBioscience, #12-0042-82; 1:100.
• Anti-CD4 (FITC): BioLegend, #100510; 1:200.
• Anti-CD8α (APC): BioLegend, #100711; 1:200.
• Anti-TCR-β (FITC): BioLegend, #109205; 1:100.
• Anti-CD62L (PE): BioLegend, #104407; 1:200.

Secondary Antibodies:
• Alexa Fluor 488-conjugated goat anti-rabbit IgG: Invitrogen, A-11034; 1:500.
• Alexa Fluor 488-conjugated goat anti-mouse IgG: Invitrogen, A-11029; 1:500.
• Alexa Fluor 594-conjugated goat anti-rabbit IgG: Invitrogen, A-11037; 1:500.
• Alexa Fluor 594-conjugated goat anti-mouse IgG: Invitrogen, A-11005; 1:500.
• HRP-conjugated anti-mouse IgG: Cell Signaling Technology, #7076S; 1:10,000.
• HRP-conjugated anti-rabbit IgG: Cell Signaling Technology, #7074S; 1:10,000.

Validation

All commercial antibodies were validated by the manufacturers for the indicated species and applications as specified on their respective websites. The customized anti-pCBP antibody (GTX90661) was validated by comparing staining patterns in colonic tissues from CBPWT and CBPAA mutant mice; the lack of signal in CBPAA tissues, where the target phosphorylation sites were mutated to alanine, confirmed the antibody's specificity.

Eukaryotic cell lines

Policy information about cell lines and Sex and Gender in Research

Cell line source(s)	WT and p53-/- HCT116 cells were kindly provided by Prof. Z.-F. Chang (National Taiwan University). Mouse colonic crypt-derived organoids were generated from 8-week-old female mice (CBPWT and CBPAA) in our laboratory as described in the Methods.
Authentication	The HCT116 cell lines were authenticated by western blot analysis to confirm p53 expression status (p53+/+ vs p53-/-). Further STR profiling was not performed. Mouse organoids were authenticated by genotype-specific markers and functional assays. Mouse colonic organoids were authenticated by genotyping of the source mice and further validated by immunofluorescence analysis of pCBP (Ser1383/1387) expression, confirming the presence or absence of the target phosphorylation site in WT and CBPAA lines, respectively.
Mycoplasma contamination	All cell lines and organoid cultures were tested and confirmed negative for mycoplasma contamination using commercial detection kits.
Commonly misidentified lines (See ICLAC register)	No commonly misidentified cell lines were used in this study.

Animals and other research organisms

Policy information about studies involving animals; ARRIVE guidelines recommended for reporting animal research, and Sex and Gender in Research

Laboratory animals	All mice were on a C57BL/6J genetic background. Female mice at 8 weeks of age were used in all experiments unless otherwise indicated. For bone marrow chimera experiments (Fig. 2), B6.SJL-Ptprcα pepcβ/BoyJ (CD45.1) and CBPAA (CD45.2) mice of both sexes were used.
Wild animals	No wild animals were used.
Reporting on sex	Female mice were primarily used to minimize gender-based physiological variations. Both sexes were included in bone marrow chimera experiments to ensure broad physiological relevance.
Field-collected samples	No field-collected samples were used.
Ethics oversight	All animal procedures were approved by the Institutional Animal Care and Use Committee (IACUC) of the College of Medicine, National Taiwan University (Approval No. (IACUC; Nos. 20170357, 20170454, 20200054, 20210223, 20220486, 20230281 and 20240106).

Note that full information on the approval of the study protocol must also be provided in the manuscript.

Plants

Seed stocks	N/A
Novel plant genotypes	N/A
Authentication	N/A

Flow Cytometry

Plots

Confirm that:

- [x] The axis labels state the marker and fluorochrome used (e.g. CD4-FITC).
- [x] The axis scales are clearly visible. Include numbers along axes only for bottom left plot of group (a 'group' is an analysis of identical markers).
- [x] All plots are contour plots with outliers or pseudocolor plots.
- [x] A numerical value for number of cells or percentage (with statistics) is provided.

Methodology

Sample preparation	For T cells, single-cell suspensions were isolated from the spleen and lymph nodes (mLNs and pLNs) as described in the Methods. For colonic stem cells, colonic crypts were isolated using 30 mM EDTA, followed by dissociation into single cells using TrypLE Express and a GentleMACS dissociator. Cells were filtered through a 40-µm strainer before staining.
Instrument	BD LSRFortessa and BD FACSAria III
Software	BD FACSDiva software was used for data acquisition; FlowJo and BD FACSDiva software were used for data analysis.
Cell population abundance	• T cell populations: Target CD4+ and CD8+ T cell subsets were clearly defined and isolated from debris through FSC-A/SSC-A gating. As shown in Supplementary Figures 9 and 10, the target populations were distinctly separated from background noise using isotype controls. A sufficient number of events were collected for each sample to ensure reliable and reproducible statistical quantification across all biological replicates.<br>• Colonic stem cells: The abundance of Lgr5-GFP+ cells was determined using a rigorous gating strategy (Supplementary Figure 11). The Lgr5-GFP+ population (P4) was clearly distinguished from the GFP-negative control population. For each sample, a sufficient number of events were recorded to ensure the statistical power of the percentage values reported in the study.
Gating strategy	Data were analyzed using BD FACSDiva and FlowJo software. Representative gating strategies for all flow cytometry experiments are provided in Supplementary Figure 9–11. Specific strategies are as follows:<br>• T cell populations: Total lymphocytes were identified by FSC-A vs. SSC-A gating to exclude debris and minimize the inclusion of non-lymphocytic cells (Supplementary Fig. 9 and 10). Specific sub-populations (CD4+ and CD8+) were gated as described in the Methods. For sub-population characterization (CD44 and CD62L), thresholds were established using isotype-matched controls to define the specific signal boundaries. While initial analyses utilized quadrant gates, representative figures in the manuscript (Supplementary Fig. 2g-h) were refined using rectangular gates for optimal cluster isolation, with consistent quantification logic applied across all samples.<br>• Colonic stem cells: The sequential gating hierarchy (P1–P5) is detailed in Supplementary Figure 11. Cells were first gated for size/granularity (P1) and subjected to doublet exclusion using sequential FSC-W vs. FSC-A (P2) and SSC-W vs. SSC-A (P3) gates to ensure single-cell analysis. Lgr5-GFP+ stem cells (P4) were identified using GFP-negative crypt cells as a negative control. Final population refinement (P5) was applied to ensure morphological homogeneity.

☒ Tick this box to confirm that a figure exemplifying the gating strategy is provided in the Supplementary Information.
